# Functional Dissection of the *Dictyostelium discoideum* Dynamin B Mitochondrial Targeting Sequence

**DOI:** 10.1371/journal.pone.0056975

**Published:** 2013-02-21

**Authors:** Amrita Rai, Nikolay Tzvetkov, Dietmar J. Manstein

**Affiliations:** Institute for Biophysical Chemistry, Hannover Medical School, Hannover, Germany; Université de Genève, Switzerland

## Abstract

Most mitochondrial proteins are nuclear encoded and synthesized in the cytosol with an N-terminal mitochondrial targeting sequence or presequence for subsequent import into mitochondria. Here, we describe the proteolytic processing and inner membrane potential-dependent translocation of a dynamin family member by the *Dictyostelium discoideum* mitochondrial import system. Our results show that the unusual *D. discoideum* dynamin B presequence is removed through a processing mechanism that is common for mitochondrial matrix proteins. We identified a minimal segment of the dynamin B presequence containing seven lysine residues. This 47-residue region is, in combination with consensus matrix protease cleavage sites, necessary and sufficient for mitochondrial targeting. The correct positioning of these lysine residues plays a critical role for the proper processing and mitochondrial import of dynamin B in *D. discoideum*. Fluorescent proteins tagged with the dynamin B presequence or presequence regions supporting mitochondrial import in *D. discoideum* are imported with similar efficiency into the mitochondrial matrix of mammalian cells, indicating that the basic mechanisms underlying mitochondrial protein import are highly conserved from amoebozoa to mammalia.

## Introduction

Mitochondria are endosymbiotic organelles of eubacterial origin that retain their genomic DNA [1]. Despite the presence of an independent mitochondrial genome, almost all mitochondrial proteins are encoded by nuclear genes that are translated in the cytoplasm and have to be translocated across mitochondrial membranes [2], [3]. Most mitochondrial proteins contain a N-terminal presequence that serves as targeting sequence for import into the mitochondrial matrix. The presequence is recognized by cytosolic chaperones of the Hsp70 family. The resulting interaction maintains the preproteins in a conformation competent for import [4]–[6]. Presequences consist usually of 10–80 amino acid residues, display a strong bias for basic, hydroxylated, and hydrophobic amino acids, and contain a region with a high propensity to form an amphipathic α-helix [7]–[9]. Transport of the preprotein into the matrix is facilitated by the translocase complexes of the mitochondrial outer (TOM) and inner (TIM) membranes [3]. Translocation depends on ATP hydrolysis and the electrochemical potential across the inner membrane (ΔΨ_m_). The presequence is usually proteolytically removed following import, as it might otherwise interfere with normal protein function [10], [11].

Cleavage of the presequences in the mitochondrial matrix is usually catalyzed by the mitochondrial processing peptidase (MPP) [12]–[14], which typically recognizes positively charged sequence regions that contain one of the following motifs: xRx↓x(S/x) (R-2 motif), xRx(Y/x)↓(S/A/x)x (R-3 motif), or xx↓x(S/x) (R-none motif) [15]–[17]. The presequence binds to the active site of the MPP in an extended conformation. In addition some presequences can be further processed by the mitochondrial intermediate protease (MIP). MIP acts after cleavage by MPP has occurred and usually recognizes the so-called R-10 motif: Rx↓(F/L/I)xx(T/S/G)xxxx↓ [18], [19]. MPP and MIP are structurally and functionally conserved across species, and their human homologues have been identified as potential players in mitochondrial diseases such as maternally inherited diabetes and deafness (DAD), neuropathy, ataxia, retinitis pigmentosa (NARP), Leigh’s syndrome and spastic paraplegia [20].

Relatively little experimental data is available for the mitochondrial import system in *D. discoideum* [21]. The experimentally verified presequences in this organism consist of an α-helix with the typical amphipathic arrangement of its opposite faces, followed by a potential MPP cleavage site. Some proteins in *D. discoideum* can be imported into mitochondria co-translationally [22], in contrast to the more common post-translational process. However, this is not related to their presequences but due to specific properties of their mRNAs.

Dynamins and dynamin-related proteins (DRPs) are large self-assembling GTPases that regulate membrane dynamics and thus participate in numerous cellular processes [23]. They have been shown to play an essential role in mitochondrial division and fusion [24], [25]. Yeast Dnm1 and mammalian Drp1 associate with mitochondrial fission sites upon oligomerization [24], [26]. It is thought that multimeric ring-like structures with dimensions similar to those of dynamin-1 multimers promote membrane scission by generating force and changing membrane curvature using the energy from GTP hydrolysis [27]–[29]. Proper mitochondrial fusion and fission are essential for normal mitochondrial function. The mammalian mitochondrial fission machinery consists of the outer membrane protein Fis1 and the dynamin-related GTPase Drp1. Three large GTPases - OPA1 and the mitofusins Mfn1 and Mfn2 are required for the fusion of inner and outer mitochondrial membranes, respectively in mammals. In yeast the OPA1 ortholog Mgm1 and the mitofusin ortholog Fzo1 play similar roles [25], [30]–[33]. Mgm1 is targeted to the inner membrane by a bipartite targeting sequence that consists of an N-terminal signal sequence followed by hydrophobic amino acid clusters. The hydrophobic clusters act as *stop-transfer sequence* that prevents further translocation across the inner membrane. This bipartite targeting sequence is processed in two cleavage steps [34], [35]. The N-terminal 9-kDa signal sequence of Mgm1 is initially cleaved by MPP and the next processing step is catalyzed by Pcp1, a protein that shares a high degree of sequence similarity with Rhomboid-type serine proteases [34].

The main aim of this study is to map the dynamin B presequence and find out the essential features required for mitochondrial targeting. *D. discoideum* dynamin B (GenBank XP_642447) is initially produced as preprotein with long amino-terminal presequence having unusually long asparagine stretch. Here, we describe the characterization of the dynamin B leader sequence. We identified a short sequence within the dynamin B presequence that can serve as mitochondrial targeting sequence (MTS). The presence of the long poly-asparagine repeat with in the presequence has no influence on the targeting efficiency of the dynamin B presequence. Moreover, our results show that the dynamin B presequence can drive the efficient import of proteins into the mitochondrial matrix of mammalian cells, indicating a highly conserved underlying mechanism.

## Materials and Methods

### Cell Culture

*D. discoideum* AX2 cells were grown in HL-5C medium (Formedium) at 21°C. Cells were transformed with expression constructs by electroporation and transformants were selected in presence of 10 µg/ml G-418 (Formedium) as described [36] and checked for expression.

Mammalian HEK293T cells were maintained in DMEM medium supplemented with 10% fetal calf serum, 2 mM L-glutamine and penicillin/streptomycin at 37°C in the presence of 5% CO_2_. For GFP expression, cells were grown in 35 mm plate until they reached 60–70% confluency and transfected transiently using polyethylenimine (PEI) (Sigma) in 6 well cell culture plates, 3∶1 PEI:DNA (12∶4 µg) ratio was used for transfection. Expression was checked 16 hr after transfection. For membrane potential experiment, 8 hr post transfection cells were treated with 10 µM Carbonyl cyanide 3-chlorophenylhydrazone (CCCP, Sigma) and expression was checked 12 hr after adding CCCP.

### Plasmid Construction

All plasmids used for expression in *D. discoideum* in this work were constructed by cloning PCR amplified DNA sequences encoding the 136 amino acid residues dynamin B presequence or fragments of it between the SacI and XbaI sites of plasmid pDXAmcsYFP [37]. In the context of the expression vectors listed below the presequence is referred to as NTS. Expression vectors for the following EYFP tagged constructs were generated : pDXA/NTSEYFP (NTS residues 1–136); pDXA/NTS ΔN1–EYFP (NTS residues 28–136); pDXA/NTS ΔN2–EYFP (NTS residues 51–136); pDXA/NTS ΔN3EYFP (NTS residues 103–136); pDXA/NTS ΔC–EYFP (NTS residues 1–112); pDXA/NTS ΔI1–EYFP (NTS residues 1–64 fused to 103–136); pDXA/NTS ΔI2–EYFP (NTS residues 28–64 fused to 103–112); and pDXA/NTS ΔI3–EYFP (NTS residues 28–50 fused to 103–112). Lysine residues have been mutated to alanine on the ΔI2 background and five different ΔI2 mutant constructs were made, pDXA/NTS ΔI2 K2A–EYFP (K 38, 41 to A), pDXA/NTS ΔI2 K5A–EYFP (K29, 40, 47, 58 and 61 to A), pDXA/NTS ΔI2 K7A–EYFP (K 29, 38, 40, 47, 58 and 61 to A), pDXA/NTS ΔI2 K38A–K40A–EYFP and pDXA/NTS ΔI2 K29A–K61A–EYFP. NTS and ΔI2 constructs lacking R-like recognition sequence (residues 103–112), pDXA/NTS ΔRS–EYFP and pDXA/NTS ΔI2 ΔRS–EYFP were made. Arginine 105 (R-motif) in the putative cleavage site is mutated to alanine to generate pDXA/NTS R105A–EYFP and pDXA/NTS ΔI2 R105A–EYFP constructs.

Mammalian expression constructs were generated in the eukaryotic expression vector pEGFP–N1 (Clontech). DNA fragments encoding the dynamin B presequence, fragments of it or mutated NTS fragments were inserted between the BamHI and XhoI sites of the vector. The resulting plasmids pEGFP–NTS, pEGFP–NTS ΔI2, pEGFP–NTS ΔRS, pEGFP–NTS R105A, pEGFP–NTS ΔI2 ΔRS, pEGFP–NTS ΔI2 R105A, pEGFP–NTS ΔI2 K2A, pEGFP–NTS ΔI2 K5A, pEGFP–NTS ΔI2 K7A, pEGFP–NTS ΔI2 K38A–K40A and pEGFP–NTS ΔI2 K29A–K61A were made.

Mutagenesis was performed as described [38] and all constructs were verified by sequencing.

### Fluorescence Microscopy

*D. discoideum* AX2 cells were grown on glass bottom plates (MatTek Corp) to 30–40% confluency. For epi-fluorescence imaging, cells were washed twice with 10 mM MES-NaOH pH 6.5, 2 mM MgCl_2_, 0.2 mM CaCl_2_ and kept in the buffer at 21°C. Images were taken at 512 nm with an Olympus 1×81 inverted microscope equipped with a 100×1.45 NA objective and a Hamamatsu C10600 ORCA R2 CCD camera. For confocal microscopy, samples were prepared as described previously [39], except that they were permeabilized for 2 min with 70% ethanol or 0.02% Triton X-100 at room temperature. Mouse monoclonal anti-mitoporin antibody 70-100-1 [40] rabbit polyclonal anti-GFP antibody AB3080 (Millipore) and appropriate Alexa conjugated secondary antibodies were used. Images were taken with a 63×1.4 NA oil objective on Leica TCS SP2 laser scanning confocal microscope. All procedures were carried out at room temperature unless otherwise stated.

Mammalian NTS-EGFP producing HEK 293T cells were incubated for 30 min with 250 nM Mitotracker Deep Red 633 (Molecular Probes) in DMEM media without serum at 37°C in the presence of 5% CO_2_ for 30 min. Cells were fixed with 4% paraformaldehyde in PBS for 15 min at room temperature. For Tom20 staining, cells were washed twice with PBS after fixation and unreacted paraformaldehyde was quenched with 100 mM glycine in PBS for 5 min. Cells were permeabilized by incubation with 0.02% Triton X-100 for 5 min, washed three times with PBS and were blocked with 0.045% fish gelatin (Sigma Aldrich) and 0.5% BSA in PBS (PBG) for one hour at room temperature, followed by overnight incubation at 4°C with rabbit Tom20 antibody (Santa Cruz) diluted (1∶150) in PBG . After extensive washing with PBS, cells were labeled for one hour at room temperature with 1∶250 dilutions of the appropriate secondary antibody conjugated with Alexa Fluor 555 (Invitrogen). After extensive washing with PBS, cover slips were mounted on glass slides with SlowFade Gold antifade reagent (Invitrogen). Images were taken with a Leica TCS SP2 confocal laser scanning microscope equipped with a 63×1.4 NA objective.

### Immunoblot Analysis

For whole cell lysates immunobloting experiments, cells were harvested, washed, lysed in buffer containing 50 mM Tris pH 7.5, 20 mM Na PPi, 20 mM NaHSO_3_, 5 mM EGTA, 5 mM EDTA, 1 mM PMSF, 1× Protease inhibitor cocktail (Roche), 0.5% Triton-X100 and mixed with an equal amount of boiling 2× SDS loading buffer. To analyse specific processing of NTS-EYFP, GFP immunoblot of isolated mitochondria was performed. Mitochondria were prepared as described [40] from AX2 cells overproducing NTS-EYFP and equal amount of boiling 2× SDS loading buffer was added. Samples were run on 15% SDS-PAGE and transferred to nitrocellulose membrane (Whatman). Membranes were blocked with PBS/ 0.05% Tween 20 containing 5% powdered skim milk, followed by incubation with mouse anti-GFP antibody (Roche). A horseradish peroxidase-conjugated secondary antibody (Thermo Scientific) was used for detection. Following development using the SuperSignal West Dura Substrate (Thermo Scientific) images were taken with a GelDoc system (Bio Rad).

### Protease Accessibility Assay

Mitochondria from transiently transfected HEK293T cells overproducing NTS-EGFP were prepared using the mitochondria isolation kit (Thermo Scientific). Purified mitochondria were incubated on ice for 10, 20, and 30 min in the presence or absence of up to 0.1 mg/ml trypsin. Reactions were stopped by the addition of 10 mM PMSF and SDS-sample buffer and heated at 90°C for 10 min; equal amounts of protein were separated by SDS–PAGE and analyzed by immuno-blotting on nitrocellulose membranes.

Monoclonal mouse anti-GFP (Roche), mouse anti-CytC (Mitoscience), rabbit anti-Tom20 (Santa Cruz) were used, Mitochondrial Hsp60 was detected using rabbit polyclonal GroEL antibody (Sigma Aldrich). To show that the processed NTS-EGFP construct is sensitive to proteolytic degradation, we performed additional trypsin digests after detergent-permeabilization of mitochondria.

### Bioinformatics

MITOPRED [41], Mitoprot II [42], Predotar [43], PSORT II [44], Subloc [45], and TargetP [46] were used to predict mitochondrial targeting sequences. Prediction of MPP and MIP cleavage sites was done with PSORT II [44] and by visual inspection. The SCRATCH [47] protein structure and structural feature prediction server prediction server was used to obtain tertiary structure models.

## Results and Discussion

### The Presequence of Dynamin B Serves as Mitochondrial Targeting Sequence

**Figure 1:**
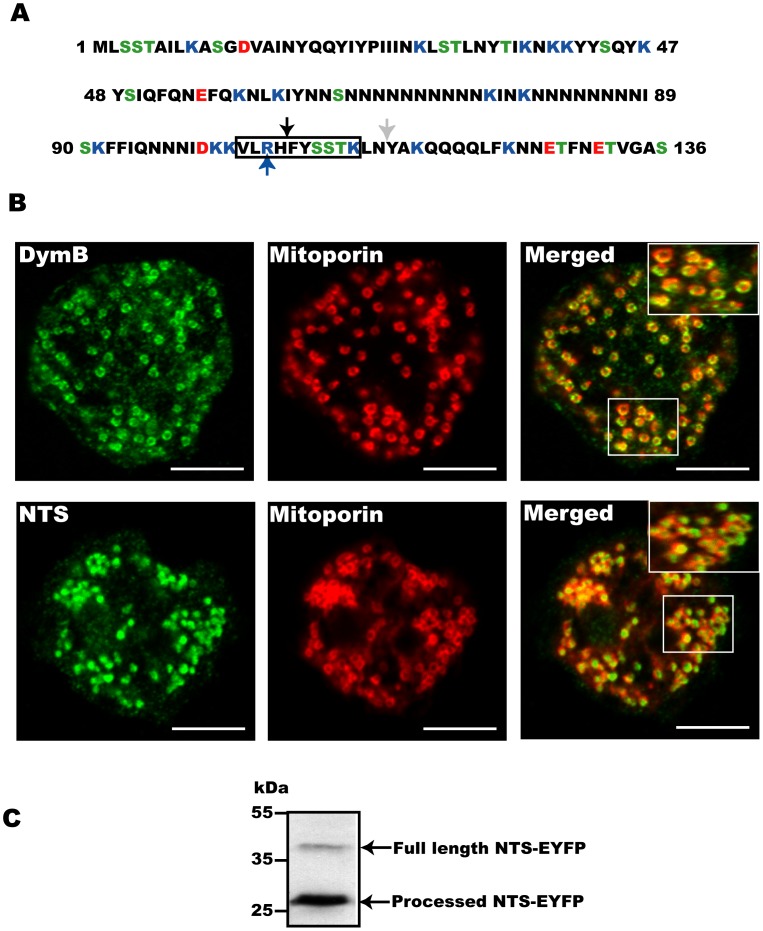
The dynamin B NTS is sufficient for mitochondrial targeting. (**A**) N-terminal 136 amino acid sequence of dynamin B. Positively charged amino acid residues are shown in blue, negatively charged residues in red, and serine and threonine residues in green. The black box marks the predicted R-like recognition sequence. Predicted MPP and MIP cleave sites are indicated by black and gray arrows, respectively. Arginine 105 is marked by a blue arrow. (**B**) The dynamin B presequence contains a functional mitochondrial targeting signal. The upper panel shows AX2 cells transformed with pDXA/DymB-EYFP (control). The lower panel shows AX2 cells transformed with pDXA/NTS-EYFP. Cells were fixed and immuno-stained as described in material and method. Single plane confocal images are shown. Scale bars, 5 µm. (**C**) The dynamin B presequence is processed upon mitochondrial translocation. Intact mitochondria from AX2 cells overproducing NTS-EYFP were purified, separated on 15% SDS-PAGE, blotted and probed with affinity purified mouse anti-GFP antibody.

*D. discoideum* dynamin B is produced as preprotein with a presequence of 136 amino acid presequence that is rich in Asn (25%), Gln (8%), Ile (10%), Lys (12%), and Tyr (8%) and Ser (8%) residues. Analysis using the SCRATCH protein structure and structural feature prediction server [47] suggests that the dynamin B 136 amino acid presequence folds into a small globular domain. It shares no apparent homology to any other protein and contains a central 24 residue segment with 21 asparagine residues (Fig. 1A). Analysis of dynamin B with various mitochondrial targeting prediction software tools gives low mitochondrial targeting probability but indicates a potential R-10 processing site at position 115 of the dynamin B presequence (Fig. 1A). Since dynamin B has no transmembrane region this rules out the possibility of membrane insertion. Fusion of the dynamin B N-terminal sequence to the amino-terminus of EYFP (NTS-EYFP) provides experimental evidence that the dynamin B presequence targets proteins efficiently to mitochondria (Fig. 1B) [39]. Further examination of confocal images showed that most of the NTS-EYFP fluorescence signal is surrounded by the outer mitochondrial membrane marker porin, indicating that the dynamin B presequence targets EYFP to an inner mitochondrial compartment. This result is further supported by experiments performed in mammalian cells (see below). In agreement with our earlier work [39], full length dynamin B-EYFP appears to be associated with the outer mitochondrial membrane (Fig. 1B). Furthermore, immunoblots of mitochondria isolated from cells producing NTS-EYFP show that NTS-EYFP undergoes mitochondrial processing and most of the protein runs as processed 27 kDa band. Additionally, a weaker band corresponding to the unprocessed 42 kDa full length protein was observed (Fig. 1C). Our results show that NTS-EYFP is efficiently processed and translocated to the inner mitochondrial compartment. Since the NTS lacks a transmembrane region and contains potential MPP and MIP cleavage sites, our results suggest that the dynamin B NTS targets EYFP to the mitochondrial matrix, where it is processed by matrix proteases.

**Figure 2:**
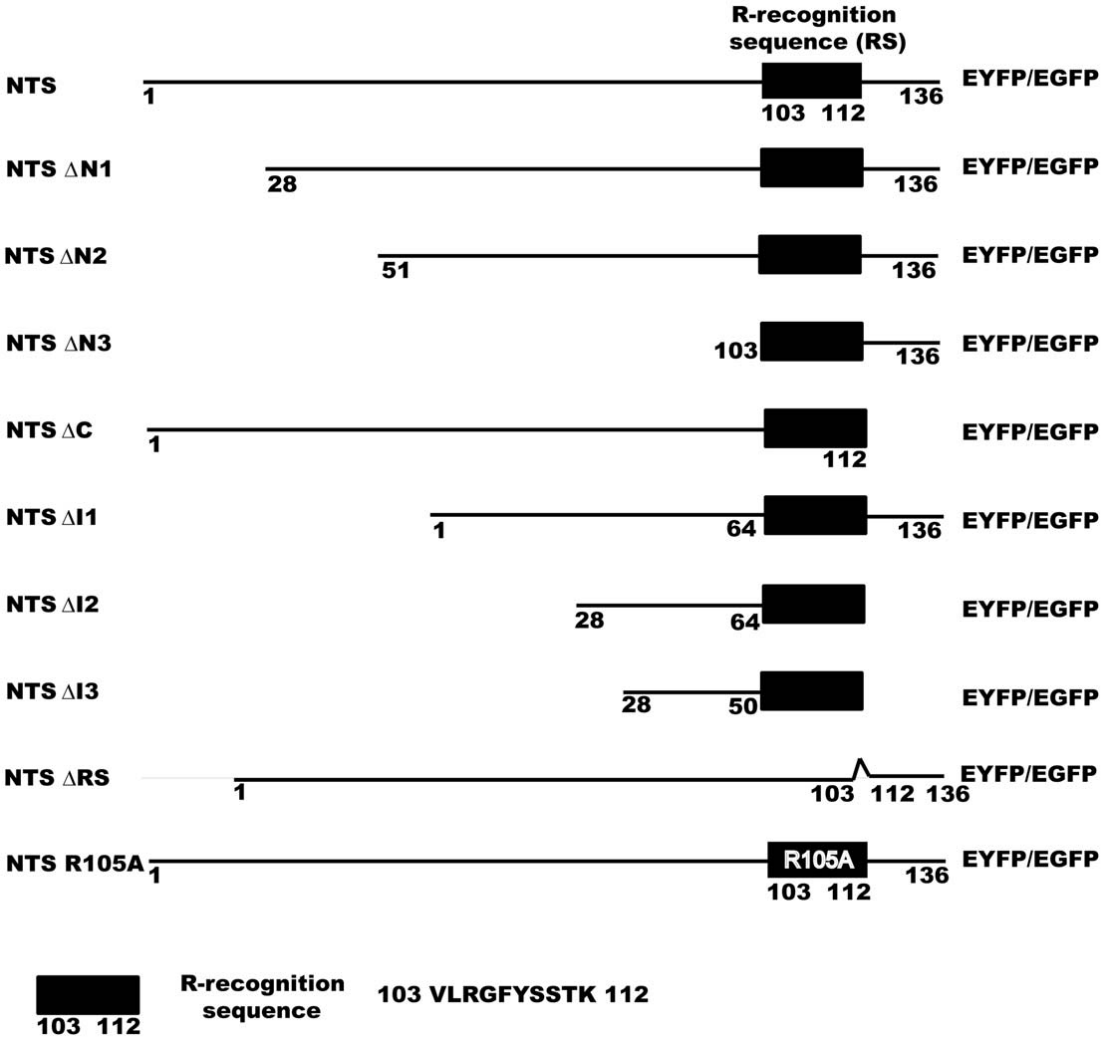
Schematic diagram of dynamin B NTS constructs. Ten different dynamin B NTS constructs are shown. Black bar represents R-like recognition sequence which comprises residues 103 to 112 of NTS.

**Figure 3:**
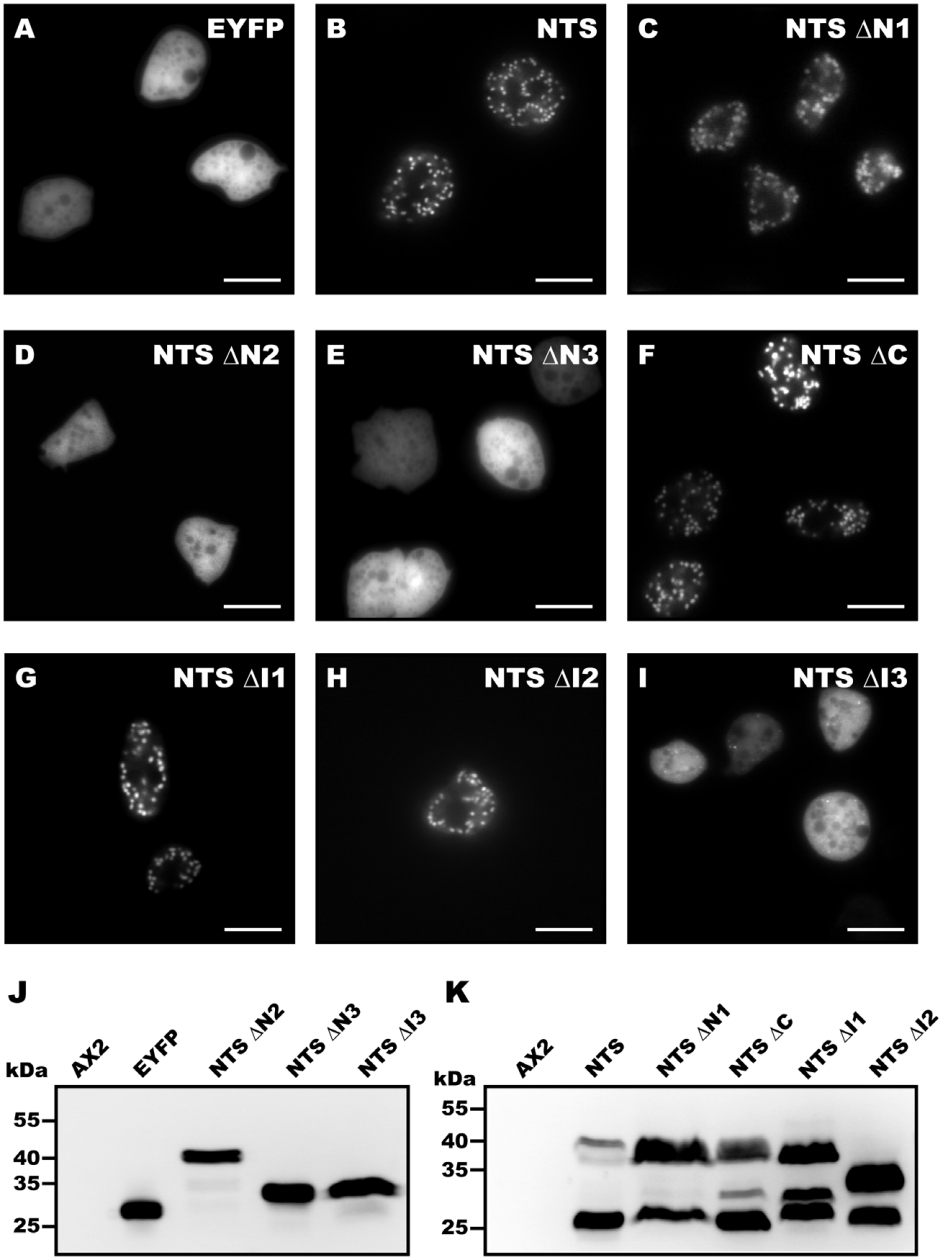
Residues 28–64 within the dynamin B presequence (NTS) are required for mitochondrial targeting. (**A–I**) AX2 cells were transformed with EYFP or with the indicated NTS fragments fused to EYFP. Images of live cells were taken by epi-fluorescence microscopy. Diffuse staining indicates cytoplasmic localization and granular staining indicates mitochondrial localization. Scale bars, 10 µm. (**J–K**) Mitochondrial targeted sequences undergo processing. (**J**) All non-targeted constructs run as a single band according to their size. GFP immuno-blot with *D. discoideum* whole cell lysates derived from untransformed cells (AX2), cells producing EYFP and EYFP-tagged constructs NTS ΔN2, NTS ΔN3 and NTS ΔI3. (**K**) GFP immuno-blot with *D. discoideum* whole cell lysates derived from untransformed cells and cells producing EYFP fusion carrying NTS, NTS ΔN1, NTS ΔC, NTS ΔI1 and NTS ΔI2 of cells is shown. Mitochondrial targeted constructs undergo processing, the upper band corresponds to unprocessed pre-protein and the lower bands correspond to the processed products showing that at least in the case of NTS ΔC and NTS ΔI1 two cleavage steps occur during processing (or alternative cleavage sites are used).

To identify the minimal region within the dynamin B presequence required for mitochondrial targeting, we generated deletion constructs in which different sub-regions of the presequence are fused to EYFP (Fig. 2). The different NTS constructs were transformed into *D. discoideum* and the distribution of YFP was analysed by fluorescence microscopy (Fig. 3). Deletion of residues 1–27 (NTS ΔN1), 113–136 (NTS ΔC) and the poly-asparagine (NTS ΔI1) stretch do not affect mitochondrial targeting (Fig. 3C, 3F, 3G and Fig. S1), whereas residues 28–64 are essential and sufficient for mitochondrial targeting. NTS ΔI2 is the smallest construct that targets YFP efficiently to mitochondria (Fig. 3H and Fig. S2A), while NTS ΔI3 is found in the cytosol (Fig. 3I). These results show that a 47-amino acid peptide consisting of dynamin B presequence residues 28–64 and residues 103–112 (R-like recognition sequence) can serve as an efficient mitochondrial targeting sequence in *D. discoideum*. Residues 28–64 are rich in positively charged, hydroxylated, and hydrophobic residues and have a high potential to form an amphipathic α-helix. These features are shared with other signals targeting proteins to the mitochondrial matrix [3]. But unlike other presequence, the dynamin B presequence contains a central asparagine-rich region. The presence of poly-asparagine repeats is quite common in *D. discoideum* proteins, but their function is not well understood [48]. In the context of this work, we suggest that the asparagine-rich region serves simply as a spacer between the minimal targeting sequence and potential protease cleavage sites and is not critical for targeting and processing. Additionaly, our results indicate that the 27 N-terminal residues of the presequence are not required for mitochondrial targeting.

As all our constructs contain potential MPP and MIP cleavage sites, we checked whether the fusion proteins undergo normal post-translational processing. Processing was not observed for non-targeted constructs NTS ΔN2, NTS ΔN3, and NTS ΔI3 (Fig. 3J). However, processing was observed for all constructs that are targeted to mitochondria. Thus, mitochondrial targeting appears to be a precondition for proteolytic removal of the NTS. Targeted constructs NTS ΔN1, NTS ΔC, NTS ΔI1, and NTS ΔI2 are proteolytically modified, although not with the same efficiency as the EYFP construct carrying the complete NTS. This appears to be linked to differences in the expression levels of the individual proteins. The presence of a third band in the lanes for NTS ΔC and NTS ΔI1 suggests that processing involves an MPP-mediated cleavage step followed by an MIP-dependent cleavage step (Fig. 3K).

### Clustering of Lysine Residues Plays an Important Role in Mitochondrial Targeting

**Figure 4:**
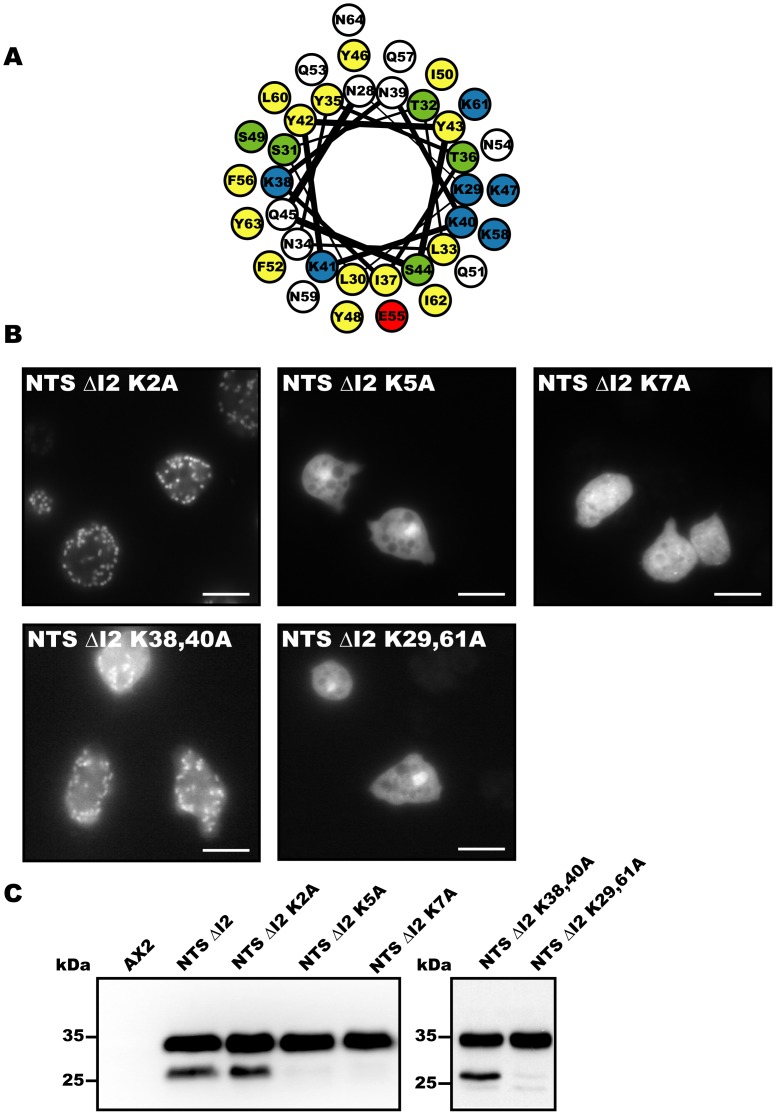
Positive charge clusters are essential for mitochondrial targeting. (**A**) Helical wheel projection of residues 28–64. Positively charged amino acid residues are shown in blue, negatively charged residues in red, serine and threonine in green and hydrophobic residues in yellow. (**B**) Localization of NTS ΔI2 mutants, NTS ΔI2–K2A, NTS ΔI2–K5A, NTS ΔI2–K7A, NTS ΔI2–K38A–K40A and NTS ΔI2–K29A–K61A. Diffuse staining indicates cytoplasmic distribution and granular staining mitochondrial targeting. Scale bars, 10 µm. (**C**) Immuno-blot loaded with *D. discoideum* whole cell lysate from untransformed cells (AX2) and cells producing EYFP-tagged NTS ΔI2, NTS ΔI2–K2A, NTS ΔI2–K5A, NTS ΔI2–K7A, NTS ΔI2–K38A–K40A, and NTS ΔI2–K29A–K61A. Mitochondrial targeted NTS ΔI2–K2A and NTS ΔI2–K38A–K40A undergo processing, while NTS ΔI2–K5A, NTS ΔI2–K7A and NTS ΔI2–K29A–K61A run as un-processed proteins.

Clustering of positive residues within the targeting sequence on one side of an amphipathic helix has been shown to be critical for specific recognition by the mitochondrial protein import machinery [49]. A helical wheel projection and an *ab initio* model of the tertiary structure of the region formed by residues 28–64 show that five of the seven lysine residues contained in the region are predicted to cluster on one side of a helix. Lysine residues 29, 40, 47, 58 and 61 lie on the same face of the α-helix, while lysine 38 and 41 are on the opposite face (Fig. 4A). Further support for the notion that the efficient translocation requires predominant clustering of positive charges on one side of the helix is provided by the behavior of the smallest construct NTS ΔI3. With a more balanced charge distribution involving three lysine residues on one face of the helix and two on the opposite face, NTS ΔI3 is not efficiently targeted to mitochondria (Fig. 3I).

To reveal the importance of individual lysine residues in the minimal targeting sequence, we mutated the lysine residues of the NTS ΔI2 construct to alanine residues. Substitution of lysine residues 38 and 41 (NTS ΔI2–K2A) with alanine did not interfere with the region’s ability to serve as an MTS (Fig. 4B and Fig. S2B). In contrast, mutation of lysine residues K29, 40, 47, 58, and 61 (NTS ΔI2–K5A) or all seven lysine residues (NTS ΔI2–K7A) led to a complete loss of mitochondrial targeting (Fig. 4B). Next, we checked whether these mutants undergo normal processing. As expected from the localization studies, ΔI2–K2A undergoes processing, while the non-targeted constructs NTS ΔI2–K5A and NTS ΔI2–K7A are not processed and run as single unprocessed band (Fig. 4C).

To establish the importance of the total number of lysine residues and the relation to their specific positions in the formation of a functional MTS, we generated two further constructs containing five lysine residues. In the first construct, we mutated lysines 38 and 40 to alanine residues, which are positioned on opposite faces of the helix (NTS ΔI2–K38A–K40A). In the second construct, we mutated lysines 29 and 61 to alanine, which are positioned on the same face (NTS ΔI2–K29A–K61A). Mutant construct NTS ΔI2–K29A–K61A is neither targeted nor processed (Fig. 4B and 4C), while the second construct NTS ΔI2–K38A–K40A with uneven charge distribution is targeted as well as processed (Fig. 4B, 4C and Fig. S2C). Thus, our results suggest that even clusters of four lysine residues are sufficient to maintain an ionic interaction between the N-terminal presequence and components of the mitochondrial import machinery, as long as the critical inbalance between the charge distribution on both faces of the helix remains established.

### Importance of the R-like Recognition Sequence for Mitochondrial Targeting and Processing

**Figure 5:**
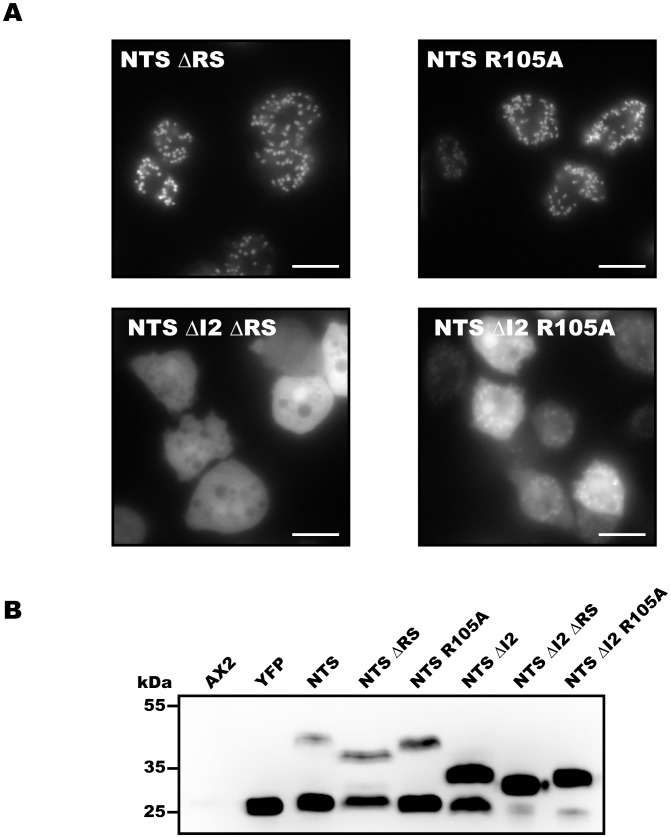
Importance of the R-like recognition sequence for mitochondrial targeting and processing. (**A**) Live cell epiflorescence imaging indicates that NTS–ΔRS and NTS–R105A are targeted to mitochondria, while NTS ΔI2–ΔRS and NTS ΔI2–R105A show less efficient mitochondrial targeting in *D. discoideum*. Scale bars, 10 µm. (**B**) Immuno-blot loaded with whole cell lysate from untransformed *D. discoideum* cells (AX2), cells producing EYFP, and EYFP-tagged constructs NTS, NTS–ΔRS, NTS–R105A, NTS ΔI2, NTS ΔI2–ΔRS, and NTS ΔI2–R105A. Similar to NTS, NTS–ΔRS and NTS–R105A undergo processing. Processing is greatly impaired in the case of NTS ΔI2–ΔRS and NTS ΔI2–R105A.

The dynamin B presequence does not contain unambiguous R-2, R-3 or R-10 sequence motif. However, an R-like recognition sequence between residues 103–112 is present, which may contain an MPP cleavage site and participate in MIP cleavage (Fig. 1A). Additionally, there may be several potential R-none sites. To understand the role of processing sites in protein localization and maturation, we generated EYFP constructs NTS–ΔRS and NTS ΔI2–ΔRS lacking the R-like recognition site. Additionally, we generated constructs NTS–R105A and NTS ΔI2–R105A, mutating the arginine residue from the putative cleavage site to alanine. Introduction of mutation R105A and deletion of the R-like recognition sequence in the full-length background do not interfere significantly with proper targeting and processing of the protein (Fig. 5A and Fig. S2D–E). However, removal or perturbation of the cleavage site leads to an almost complete loss of mitochondrial targeting and processing in the context of the NTS ΔI2 construct (Fig. 5). Construct NTS ΔI2 ΔRS is poorly processed and not targeted, while construct NTS ΔI2 R105A is processed similar poorly but targeted with reasonable efficiency. These results suggest the presence of one or more additional processing sites that are affected by the removal of the RS-region. The presence of additional R-none MPP cleavage sites within the presequence can provide added flexibility to the processing machinery during mitochondrial import.

### The Dynamin B Presequence Functions as an MTS in Mammalian Cell

**Figure 6:**
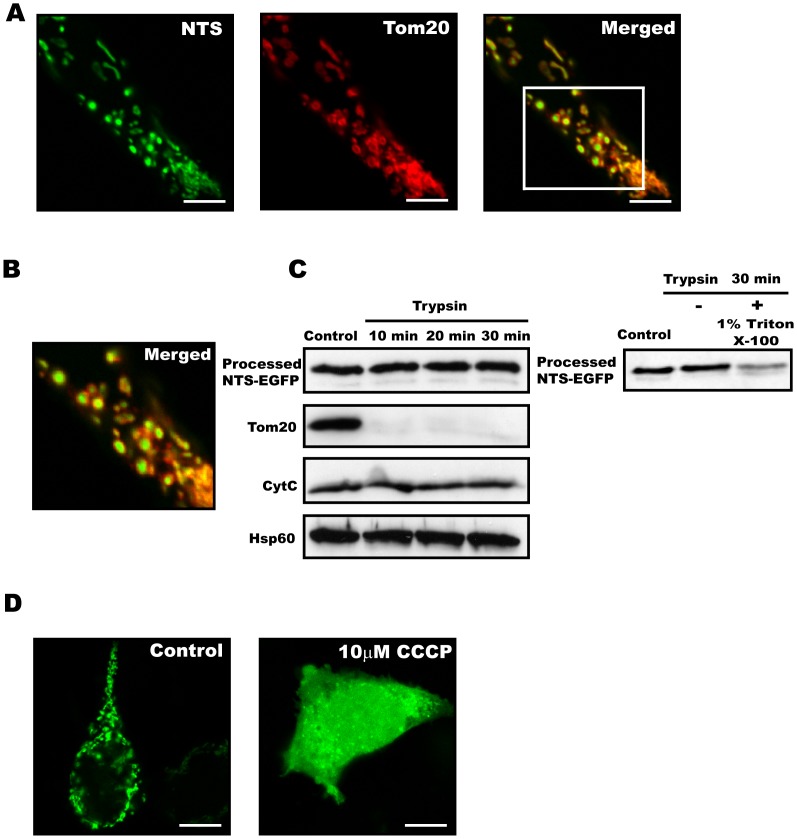
The dynamin B presequence serves as mitochondrial targeting sequence in mammalian cells. (**A**) HEK 293T cells producing pNTS-EGFP (green) and the outer membrane marker protein Tom20 (red). Scale bars, 10 µm. (**B**) The enlarged merged picture shows that that EGFP signal is surrounded by Tom20 fluorescence. (**C**) Intact mitochondria from HEK293T cells overexpressing NTS-EGFP were purified, treated with trypsin on ice, separated on 15% SDS-PAGE, blotted and probed with affinity purified mouse anti-GFP antibody, rabbit anti-Tom20, mouse anti-CytC, or rabbit anti-GRoEL. The outer membrane protein Tom20 is digested while NTS-EGFP and the inner membrane protein CytC are unaffected. The matrix protein Hsp60 was used as loading control. To show that processed NTS-EGFP can be digested, detergent**-**permeabilized mitochondria were trypsin treated. (**D**) Mitochondrial import by the dynamin B presequence requires an intact mitochondrial inner membrane membrane potential ΔΨ_m_. 10 µM CCCP was added to the cell culture medium 8 hrs post transfection. Next, cells were incubated for 12 hrs in the presence of the H^+^ -ionophore before fixation.

In order to determine whether the unusual dynamin B presequence can serve as mammalian mitochondrial targeting sequence, we generated mammalian expression vectors where the dynamin B presequence or sequences derived from it are fused to the N-terminus of EGFP. Transfection of the NTS-EGFP construct into HEK293T cells results in the production, mitochondrial targeting, and proteolytic processesing of the protein. Confocal images show that the NTS-EGFP signal is surrounded by the fluorescence signal from the outer membrane protein Tom20, indicating that the protein is targeted to an inner mitochondrial compartment. This result is further supported by protease accessibility experiments. Here, the outer mitochondrial membrane marker Tom20 is digested, while the processed GFP construct is apparently protected from degradation due to its location in the mitochondrial matrix protected. Control experiment using detergent-permeabilized mitochondria show that the processed GFP construct is degraded when direct contact with trypsin is established (Fig. 6A–C).

The TIM complex-dependent import of proteins into the mitochondrial matrix requires energy provided by the mitochondrial inner membrane potential ΔΨ_m_, whereas proteins targeted to the other mitochondrial compartments do not [3]. To test whether the transport of NTS-tagged proteins to the mitochondrial matrix is ΔΨ_m_-dependent, we over-expressed NTS-EGFP in HEK293T cells. Transformed cells that were treated with the H^+^ -ionophore CCCP showed a marked increase in the ratio of cytoplasmic to mitochondrial matrix localization of the NTS-EGFP construct compared to an untreated control. This observation provides support for the concept that the translocation of NTS-EGFP to the mitochondrial matrix is mediated by a ΔΨ_m_-dependent mechanism (Fig. 6D). The residual mitochondrial signal can be explained by mitochondrial import prior to the CCCP treatment.

Results describing the localization and processing of constructs carrying an altered version of the NTS in HEK293T cells are similar to those obtained for *D. discoideum*. Constructs NTS ΔRS and NTS–R105A are targeted to mitochondria and proteolytically processed (Fig. S3). Multiple bands were observed on immuno-blots when lysates from HEK293T cells expressing these proteins were analysed (Fig. S3E). This might be due to the combined effects of MPP and MIP or differences in specificity compared to their *D. discoideum* counterparts.

The minimal construct (NTS ΔI2) is properly processed and targeted to mitochondria in HEK293T cells. Processing of ΔI2 in HEK 293T cells appears to be more efficient than in *D. discoideum* and gives similar products. Deletion of the R-recognition site or introduction of mutation R105A reduces the targeting efficiency of NTS ΔI2 (Fig. S4A–C). Compared to the situation in *D. discoideum*, NTS ΔI2 ΔRS and NTS ΔI2 R105A are more completely processed in HEK 293T cells (Fig. S4D). However, we cannot exclude that the unspecific action of cytosolic proteases contributes to the processing.

To test the importance of key lysine residues for mitochondrial targeting in mammalian cells, we transfected HEK293T cells with NTS ΔI2–K2A, NTS ΔI2–K5A, NTS ΔI2–K7A, NTS ΔI2–K38A–K40A and NTS ΔI2–K29A–K61A constructs. Again, we observed similar results compared to the situation in *D. discoideum*. NTS ΔI2–K2A and NTS ΔI2–K38A–K40A are targeted to mitochondria, while NTS ΔI2–K5A, NTS ΔI2–K7A and NTS ΔI2–K29A–K61A are not targeted (Fig. S5). The non-targeted NTS ΔI2–K5A, NTS ΔI2–K7A and NTS ΔI2–K29A–K61A constructs display the same electrophoretic mobility as EGFP. This is most likely the result of nonspecific proteolytic degradation by cytosolic proteases that cleave off the exposed unfolded preprotein region from the tightly folded EGFP core (Fig. S6).

In summary, our data show that the dynamin B presequence serves as an efficient targeting sequence in the ΔΨ_m_-dependent translocation of proteins from the cytosol into the mitochondrial matrix. The asparagine-rich region in the central part of the dynamin B presequence separates import sequences from processing sequences and does not seem to play a role in mitochondrial localization. Our results define a minimal sequence formed by residues 28 to 64 that, in combination with mitochondrial protease cleavage sites, is sufficient for efficient protein targeting to mitochondria and proteolytic processing. The presence of a cluster of lysine residues on one side of the amphipathic helix is a key feature of the mitochondrial targeting sequence. Four properly positioned lysine residues are sufficient for effective targeting of a minimal construct. We demonstrate that the underlying mechanism of protein translocation from the cytoplasm into the mitochondrial matrix is evolutionarily conserved from social amoebae to humans.

## Supporting Information

Figure S1 **Mitochondrial localization of dynamin B presequence deletion constructs. (A)** Cells transformed with NTS ΔN1, **(B)** NTS ΔC and **(C)** NTS ΔI1 are shown. Cells were co-stained with mitoporin. Scale bars, 5 µm.
(TIF)

Figure S2 **Mitochondrial localization of dynamin B presequence mutant constructs. (A)** Cells transformed with NTS ΔI2, **(B)** NTS ΔI2 K2A, **(C)** NTS ΔI2 K38A-K40A, **(D)** NTS ΔRS and **(E)** NTS R105A are shown. Cells were co-stained with mitoporin. Scale bars, 5 µm.
(TIF)

Figure S3 **The dynamin B presequence targets EGFP to mitochondria in mammalian cells.** **(A)** HEK293T cells transfected with pEGFP (control), **(B)** pEGFP**–**NTS, **(C)** pEGFP**–**NTS ΔRS and **(D)** pEGFP**–**NTS R105A are shown. Cells were live-stained with Mitotracker Alexa 633 and subsequently fixed. Scale bars, 10 µm. **(E)** Immuno-blot loaded with whole cell lysate from untransfected HEK293T cells and HEK293T cells producing EGFP and EGFP-tagged constructs NTS, NTS ΔRS, and NTS R105A. Constructs NTS, NTS ΔRS, and NTS R105A are completely processed.
(TIF)

Figure S4 **Importance of R-like recognition sequence in mitochondrial targeting and processing in mammalian cells.** Deletion of the R-like recognition site or mutation of R105A in the context of the ΔI2 construct leads to a significant decrease in mitochondrial targeting. **(A)** HEK293T cells transfected with pEGFP**–**NTS ΔI2, **(B)** pEGFP**–** NTS ΔI2–ΔRS, and **(C)** pEGFP**–** NTS ΔI2–R105A are shown. Cells were live-stained with Mitotracker Alexa 633 and subsequently fixed. Scale bars, 10 µm. **(D)** Immuno-blot loaded with HEK293T whole cell lysates from untransfected cells, cells producing EGFP, and cells producing EGFP-tagged constructs NTS ΔI2, NTS ΔI2–ΔRS, and NTS ΔI2–R105A. The upper band corresponds to the unprocessed protein, while the lower band corresponds to the processed protein.
(TIF)

Figure S5 **Clustered lysine residues are important for mitochondrial targeting in mammalian cells.** **(A)** HEK293T cells transfected with pEGFP–NTS ΔI2 K2A, **(B)** pEGFP–NTS ΔI2 K5A, **(C)** pEGFP–NTS ΔI2 K7A, **(D)** pEGFP–NTS ΔI2 K38A–K40A and **(E)** pEGFP–NTS ΔI2 K29A–K61A are shown. Cells were live-stained with Mitotracker Alexa 633 and subsequently fixed. Scale bars, 10 µm.
(TIF)

Figure S6 **Immuno-blot of HEK293T cells.** Whole cell lysates from untransfected cells, cells producing EGFP, and cells producing EGFP fused to NTS ΔI2, NTS ΔI2–K2A, NTS ΔI2–K5A, NTS ΔI2–K7A, NTS ΔI2–K29A–K61A, and NTS ΔI2–K38A–K40A are shown.
(TIF)
