# Supplementary figures and images for: Functional Dissection of the Dictyostelium discoideum Dynamin B Mitochondrial Targeting Sequence
Source: PLoS One. 2013 Feb 21;8(2):e56975. doi: 10.1371/journal.pone.0056975 (PMC3578813; doi:10.1371/journal.pone.0056975)

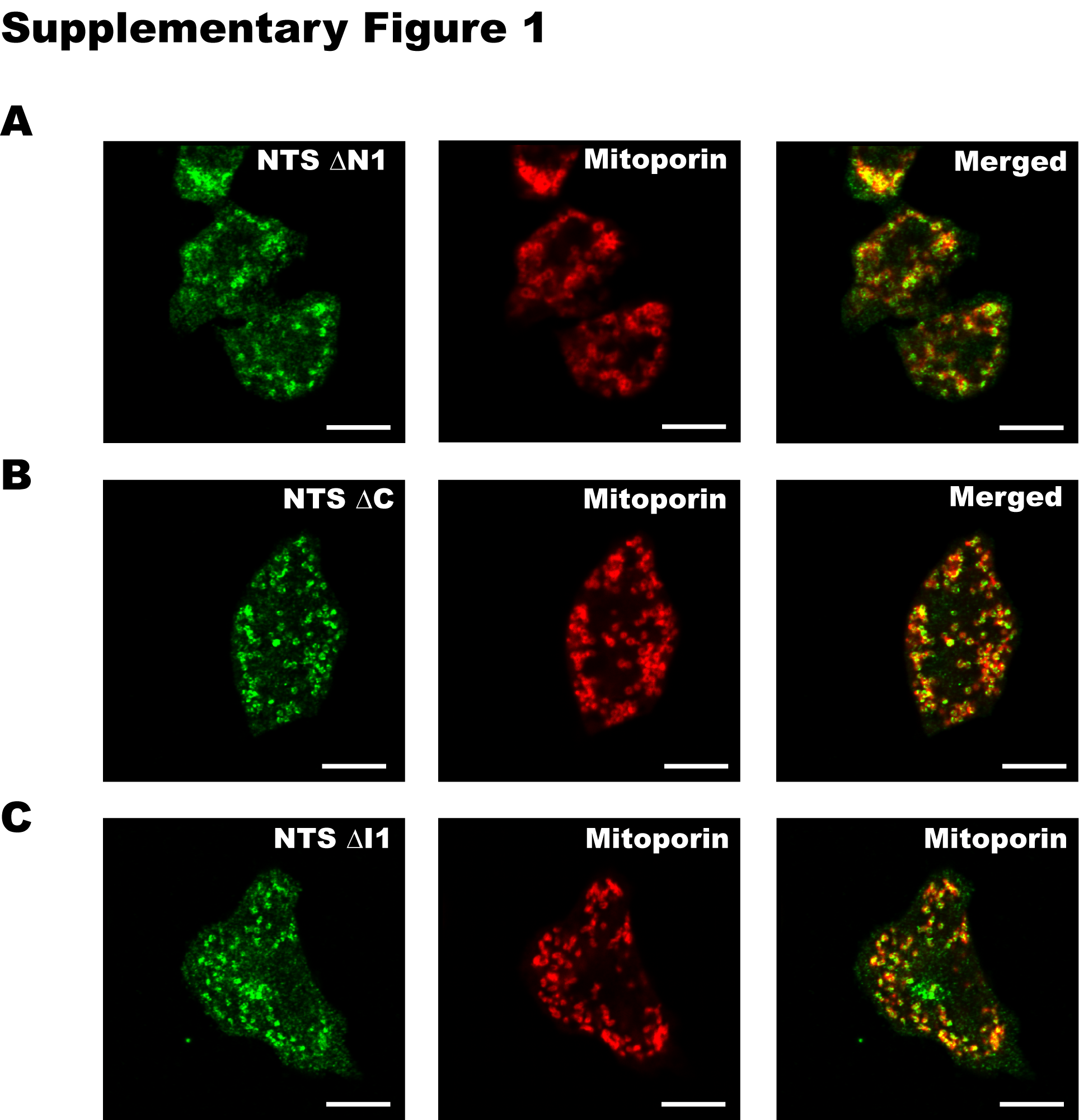

Supplement: Figure S1 — Mitochondrial localization of dynamin B presequence deletion constructs. (A) Cells transformed with NTS ΔN1, (B) NTS ΔC and (C) NTS ΔI1 are shown. Cells were co-stained with mitoporin. Scale bars, 5 µm. (TIF) [file pone.0056975.s001.tif]

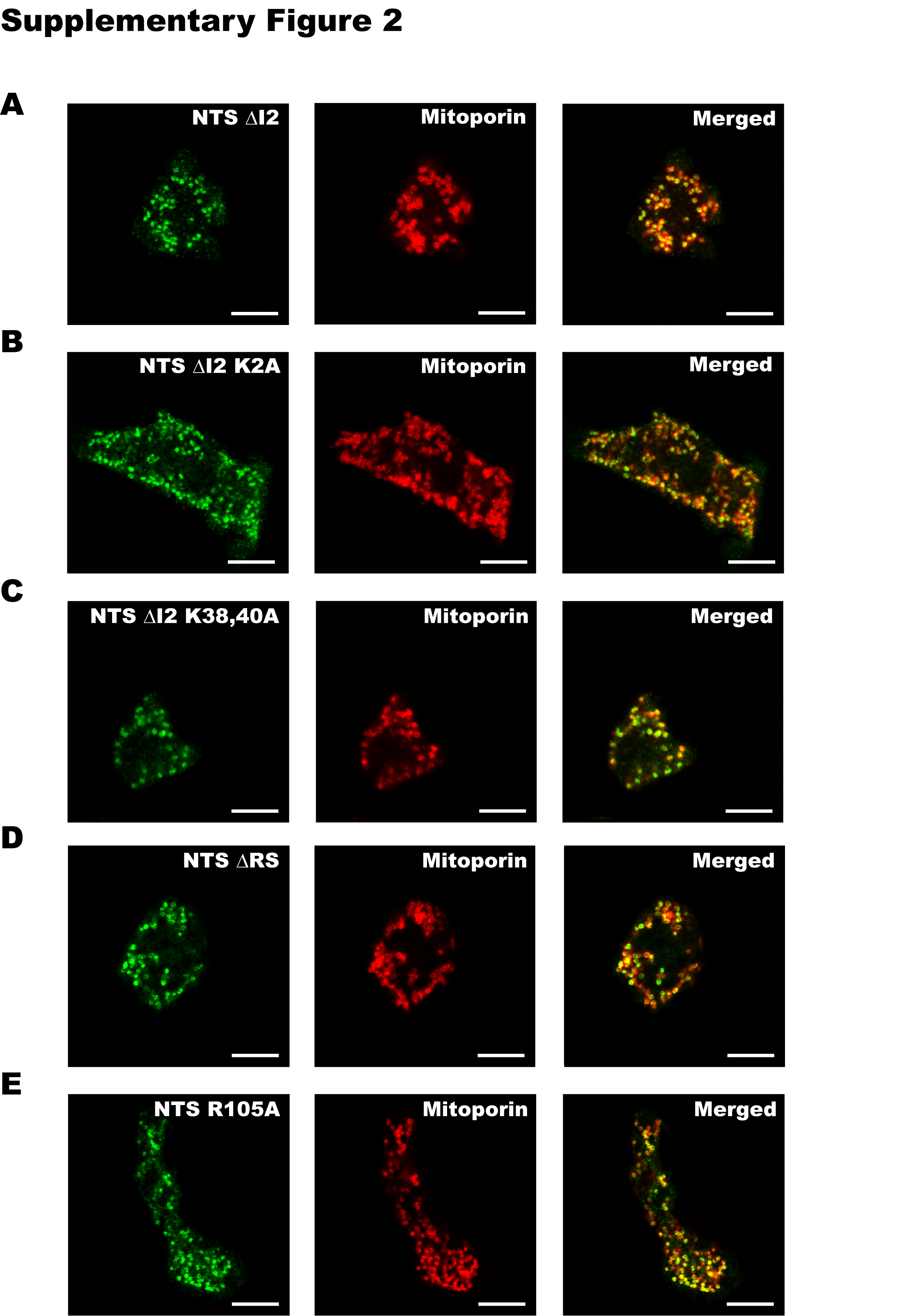

Supplement: Figure S2 — Mitochondrial localization of dynamin B presequence mutant constructs. (A) Cells transformed with NTS ΔI2, (B) NTS ΔI2 K2A, (C) NTS ΔI2 K38A-K40A, (D) NTS ΔRS and (E) NTS R105A are shown. Cells were co-stained with mitoporin. Scale bars, 5 µm. (TIF) [file pone.0056975.s002.tif]

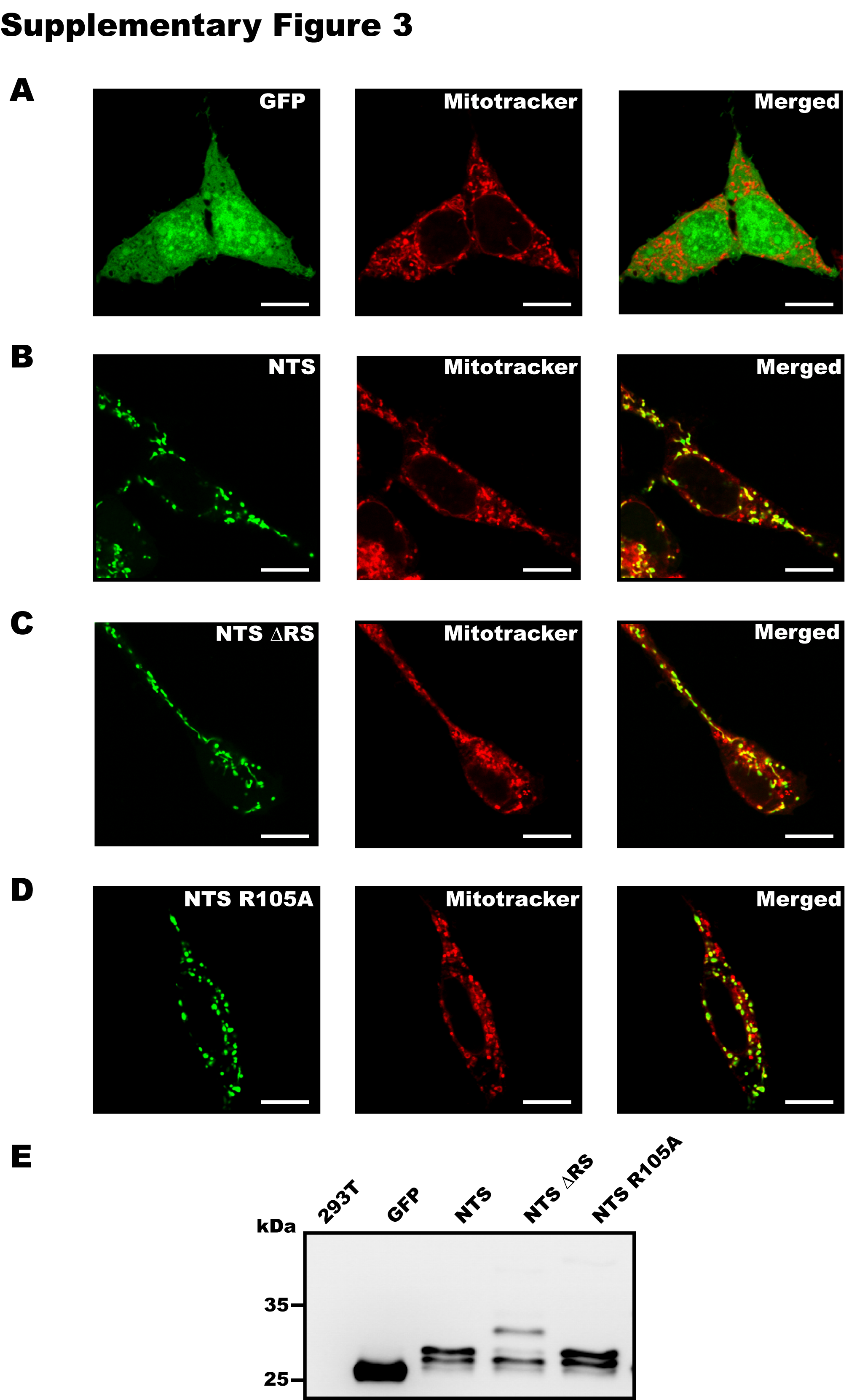

Supplement: Figure S3 — The dynamin B presequence targets EGFP to mitochondria in mammalian cells. (A) HEK293T cells transfected with pEGFP (control), (B) pEGFP–NTS, (C) pEGFP–NTS ΔRS and (D) pEGFP–NTS R105A are shown. Cells were live-stained with Mitotracker Alexa 633 and subsequently fixed. Scale bars, 10 µm. (E) Immuno-blot loaded with whole cell lysate from untransfected HEK293T cells and HEK293T cells producing EGFP and EGFP-tagged constructs NTS, NTS ΔRS, and NTS R105A. Constructs NTS, NTS ΔRS, and NTS R105A are completely processed. (TIF) [file pone.0056975.s003.tif]

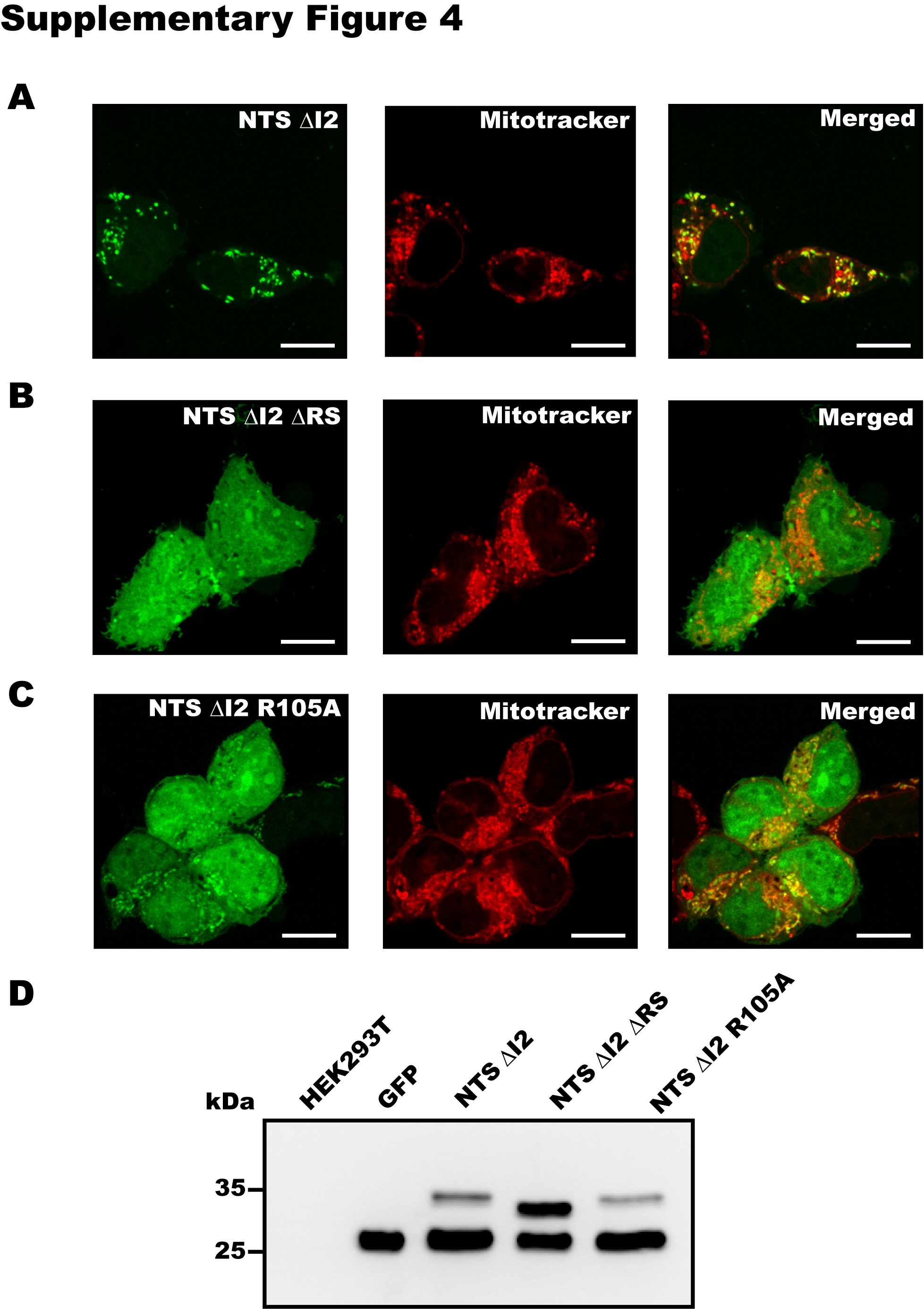

Supplement: Figure S4 — Importance of R-like recognition sequence in mitochondrial targeting and processing in mammalian cells. Deletion of the R-like recognition site or mutation of R105A in the context of the ΔI2 construct leads to a significant decrease in mitochondrial targeting. (A) HEK293T cells transfected with pEGFP–NTS ΔI2, (B) pEGFP– NTS ΔI2–ΔRS, and (C) pEGFP– NTS ΔI2–R105A are shown. Cells were live-stained with Mitotracker Alexa 633 and subsequently fixed. Scale bars, 10 µm. (D) Immuno-blot loaded with HEK293T whole cell lysates from untransfected cells, cells producing EGFP, and cells producing EGFP-tagged constructs NTS ΔI2, NTS ΔI2–ΔRS, and NTS ΔI2–R105A. The upper band corresponds to the unprocessed protein, while the lower band corresponds to the processed protein. (TIF) [file pone.0056975.s004.tif]

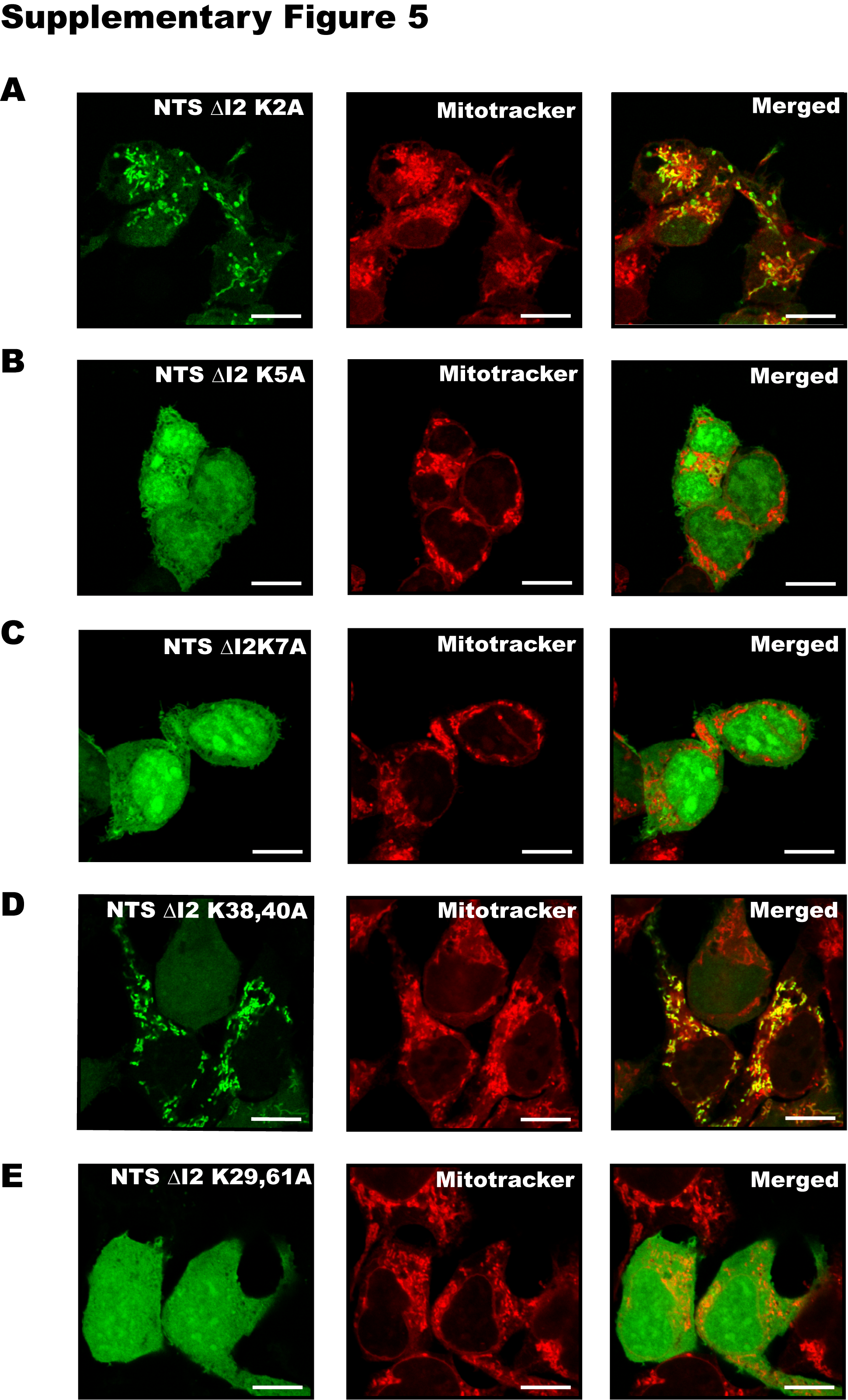

Supplement: Figure S5 — Clustered lysine residues are important for mitochondrial targeting in mammalian cells. (A) HEK293T cells transfected with pEGFP–NTS ΔI2 K2A, (B) pEGFP–NTS ΔI2 K5A, (C) pEGFP–NTS ΔI2 K7A, (D) pEGFP–NTS ΔI2 K38A–K40A and (E) pEGFP–NTS ΔI2 K29A–K61A are shown. Cells were live-stained with Mitotracker Alexa 633 and subsequently fixed. Scale bars, 10 µm. (TIF) [file pone.0056975.s005.tif]

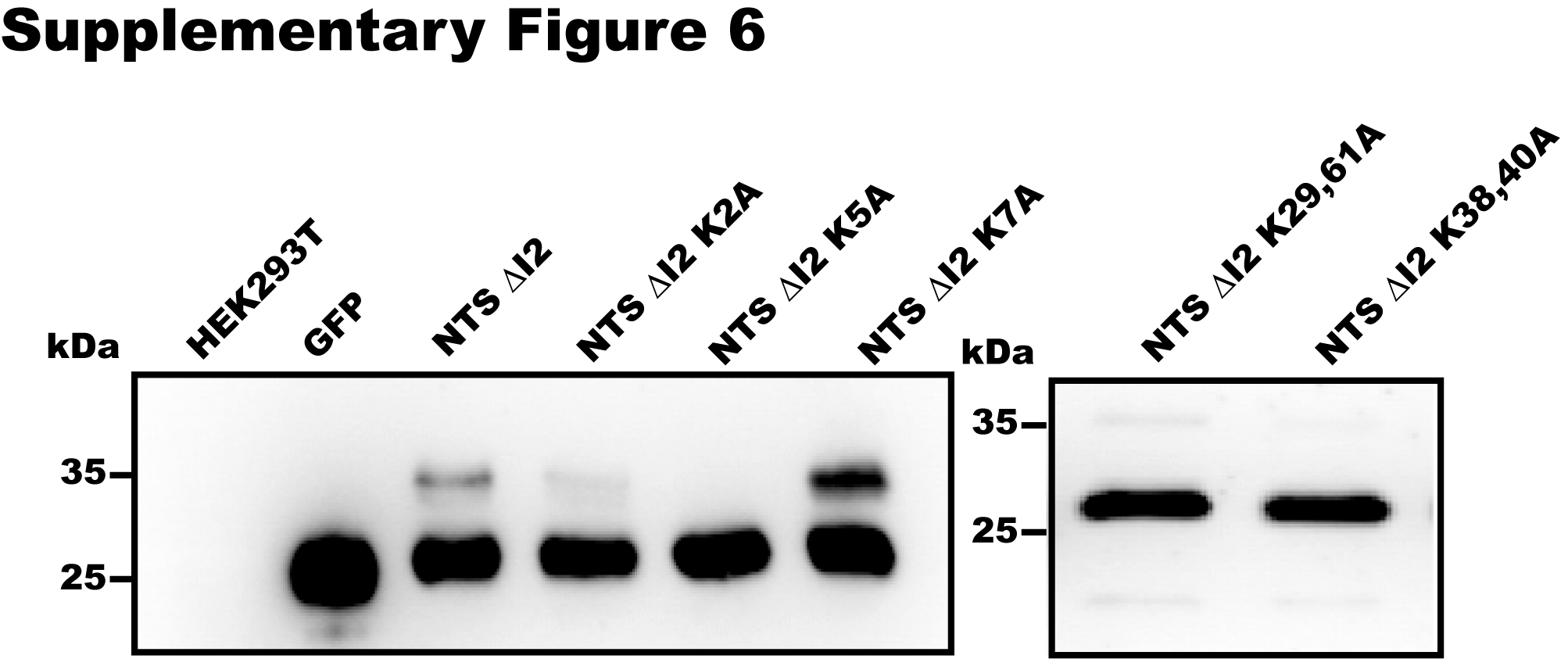

Supplement: Figure S6 — Immuno-blot of HEK293T cells. Whole cell lysates from untransfected cells, cells producing EGFP, and cells producing EGFP fused to NTS ΔI2, NTS ΔI2–K2A, NTS ΔI2–K5A, NTS ΔI2–K7A, NTS ΔI2–K29A–K61A, and NTS ΔI2–K38A–K40A are shown. (TIF) [file pone.0056975.s006.tif]
